# The Tibiofibular Line: A Reliable Method of Syndesmosis Assessment in Certain Fibula Morphologies

**DOI:** 10.7759/cureus.36300

**Published:** 2023-03-17

**Authors:** Anthony A Pollizzi, Joseph G Monir, Mollie Lagrew, Christopher Reb

**Affiliations:** 1 Department of Orthopaedics and Rehabilitation, University of Florida College of Medicine, Gainesville, USA; 2 Department of Opthalmology, University of Florida College of Medicine, Gainesville, USA; 3 Department of Orthopedic Surgery, Penn State College of Medicine, Hummelstown, USA

**Keywords:** ankle trauma, reliability, tibiofibular line, syndesmosis, ankle fracture

## Abstract

Background

The tibiofibular line (TFL) technique was initially proposed to assess syndesmosis reduction. Clinical utility was limited by low observer reliability when applied to all fibulas. This study aimed to refine this technique by describing TFL’s applicability to various fibula morphologies.

Methods

Three observers reviewed 52 ankle CT scans. Observer consistencies for TFL measurement, anterolateral fibula contact length, and fibula morphology were assessed using intraclass correlation (ICC) and Fleiss’ Kappa.

Results

TFL measurement and fibula contact length intra-observer and inter-observer consistencies were excellent (minimum ICC, 0.87). Fibula shape categorization intra-observer consistency was substantial to almost perfect (Fleiss’ Kappa, 0.73 to 0.97). Six to 10 mm of fibula contact length corresponded to excellent TFL distance consistency (ICC, 0.80 to 0.98).

Conclusion

The TFL technique appears best for patients with 6 mm to 10 mm of straight anterolateral fibula. Sixty-one percent (61%) of fibulas featured this morphology, indicating most patients may be amenable to this technique.

## Introduction

An ankle syndesmosis injury occurs in 1%-18% of ankle sprains [1]. It most commonly results from ankle external rotation or abduction occurring while the limb is loaded by body weight, which results in abnormal ankle kinematics. Recognition and fixation of unstable syndesmosis injuries are critical to preventing long-term complications. However, proper anatomic syndesmosis reduction has proven deceptively challenging to reliably achieve and has been the focus of recent literature [2].

For injuries not amenable to conservative treatment, operative reduction of the syndesmosis typically yields excellent functional outcomes when the syndesmosis is correctly reduced. Current intraoperative strategies for assessing syndesmosis reduction accuracy include the use of fluoroscopy, comparison of images of the contralateral ankle, and direct visualization of the position of the fibula relative to the tibia. However, accurate reduction of ankle syndesmosis is difficult to achieve, with the reported rate of ankle syndesmosis malreduction ranging from 0% to 52% [1,3].

Thordarson et al. examined syndesmosis malreduction in the context of joint contact stress [4]. They found that displacement decreases the contact area between the tibia and talus, shifting contact pressures from the medial quadrant of the talar dome to the mid-lateral and posterolateral quadrants. Additionally, greater degrees of displacement were generally associated with increased contact pressures, with the highest stress occurring in combinations of fibula shortening and external rotation or lateral shift. These irregular loading distributions are relevant due to their role as a risk factor for early degenerative changes and long-term osteoarthritis [4,5].

Gifford and Lutz reviewed axial ankle CT scan images at 10 mm above the plafond and observed a straight line connecting the anterolateral surface of the fibula with the anterolateral tubercle of the tibia (termed the “tibiofibular line” or TFL) at the level of the ankle syndesmosis [6]. In a series of uninjured patients with imaging that was obtained for non-ankle related injuries corroborated with normal ankle radiographic parameters, 77% (n=115) had a TFL displacement of 0 mm from the anterior tibial tubercle, 19% (n=29) had a TFL displacement of 1 mm, and 4% (n=6) were 2 mm from the tubercle. In an abnormal series of 30 patients with ankle-related injuries and abnormal radiographic syndesmosis parameters, the TFL was consistently displaced anteriorly between 4 and 19 mm.

A computed tomography (CT) scan is the gold standard for syndesmosis reduction assessment. However, intraoperative CT is not widely available, and its incorporation is cost prohibitive. While postoperative CT is available, any reduction revision at this phase requires the additional cost and morbidity of a second surgery. Of the many CT-based techniques so far described, the “tibiofibular line” technique described by Gifford and Lutz appears the most appropriate to be translated into an intraoperative technique performed under direct visualization [6].

Reb et al. demonstrated the clinical feasibility of translating the TFL into a clinical technique [7]. In follow-up work, the investigators also demonstrated excellent to near-perfect observer, intra-observer, and inter-observer reliability across several states of known syndesmosis displacement. However, this study raised questions regarding several potentially important confounding effects, including that of normal human syndesmosis anatomic variability. As Gifford and Lutz described, the anterolateral fibula was not always a perfectly flat cortical surface, but the anterior edge of the fibula and lateral edge of the tibia were connected by a smooth transition [6]. This variation in fibula morphology likely accounts for the relatively low intra- and inter-observer reliability initially reported by Reb et al. [7]. We hypothesized that these values were skewed by patients with certain fibula morphologies, which do not lend themselves to this technique, and there exists a subset of fibula morphologies for which this technique could reliably be applied. The present study evaluated the validity and reliability of the TFL as it relates to variation in fibula morphology.

The specific aims of this study were to characterize the anterolateral fibula cortical bone variance observed in the population, to describe the reliability of TFL measurements, and to describe the relationship between anatomic morphological differences and observer reliability when utilizing the TFL technique. If, as we hypothesized, certain fibula morphologies have higher intra- and inter-observer reliability, surgeons could easily identify those patients for whom this simple and cost-free intraoperative technique could be utilized.

## Materials and methods

This was a University of Florida IRB-approved (IRB201801721), retrospective, cross-sectional, quantitative, and qualitative descriptive study that utilized computed tomography in order to survey fibula morphology and determine TFL measurements. The study population included patients at a single, large tertiary referral academic hospital who have undergone foot and ankle CT scans for injuries of the foot. Inclusion criteria involved patients with ankle CTs that were imaged at least 30 mm superior to the tibial plafond and were radiographically without signs of injury or past operative treatment of the ankle or syndesmosis. Exclusion criteria included patients with malaligned syndesmoses, ankle fractures, previous ankle operations, and CTs that are not imaged at least 30 mm above the tibial plafond.

The imaging software used in the study was Visage 7 (Visage Imaging, Inc., San Diego, CA). Each patient’s identity was confirmed using their unique medical record number, and their ankle CT was located and manually verified to meet our inclusion criteria. The image was entirely de-identified by exporting the CT into a separate teaching file within Visage and labeled with a Roman numeral corresponding to the order in which the subjects were recruited. Magnification was calibrated such that the 10 mm digital calibration on the software interface matched 10 mm on a physical ruler. This served as the standardized magnification for recording all of the data for each CT. The same magnification was maintained for all measurements within the subject. All measurements were made on axial cuts 10 mm proximal to the plafond.

**Figure 1:**
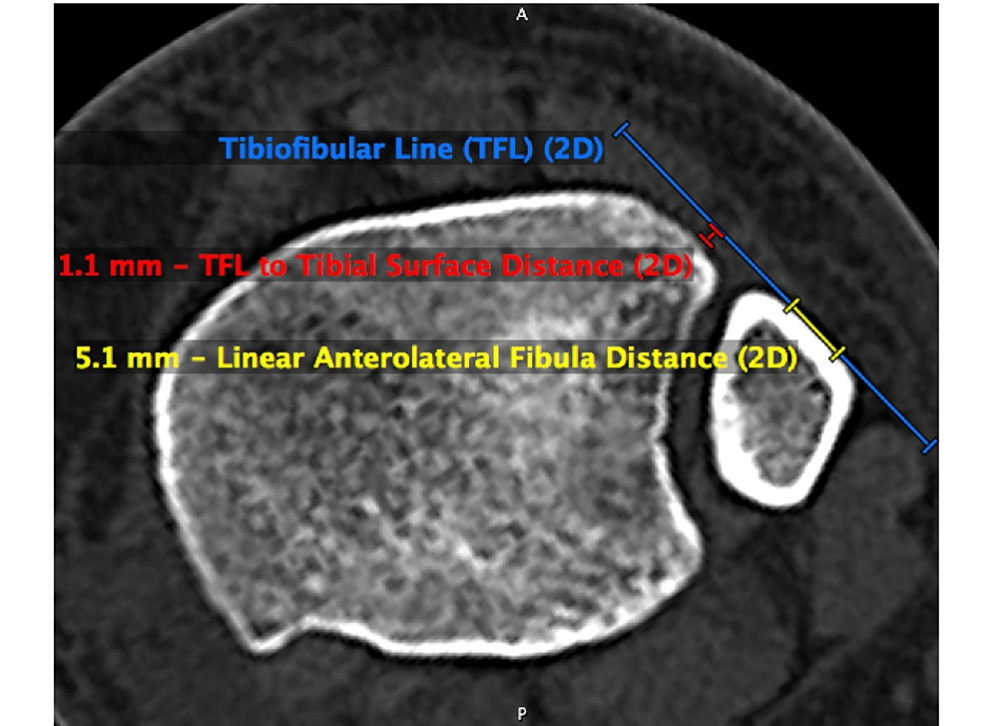
Illustration of the tibiofibular line (TFL) On axial CT 10 mm superior to the tibial plafond, the length of the linear fibula surface is measured as the linear anterolateral fibula distance. The tibiofibular line (TFL) is drawn as a straight line tangent to the flat anterolateral surface of the fibula. The CT-TFL measurement is the distance between the TFL and the anterolateral surface of the distal tibia.

**Figure 2:**
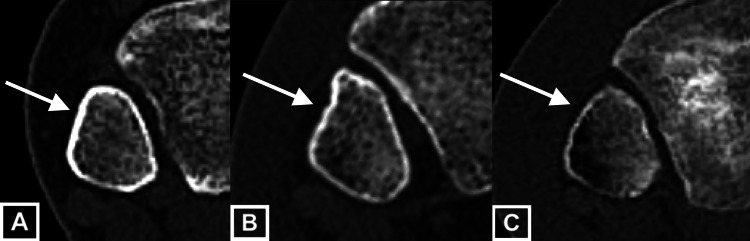
Fibular morphologies CT scan slices of different fibulas demonstrating (a) linear fibula morphology, (b) concave fibula morphology, and (c) convex fibula morphology

Anterolateral fibula morphology was categorized as linear, convex, or concave. The longest linear distance of the anterolateral fibula surface was measured (Figure 1). Morphology was subjectively categorized by each observer as linear if the fibula surface was determined to be flat (Figure 2a), concave if it did not meet the linear definition and was inwardly bowed (Figure 2b), and convex if it did not meet the linear definition and was outwardly bowed (Figure 2c).

The TFL was drawn tangential to the longest continuous linear segment on the anterolateral surface of the fibula. If there was no discernible linear surface, the line was drawn along the least concave or convex segment of the fibula cortex. A line perpendicular to this tangential line was drawn to the closest point of the tibial surface. The length of this new line was measured and recorded. In the event of a TFL transecting the tibia, the measurement was made from TFL to the furthest tibial surface point and recorded as a negative value. Three separate observers performed the measurements, and each observer repeated the measurements two more times for a total of three measurements per observer per patient. Each repeated measurement was separated in time from the prior to minimize bias due to short-term recall. Measurements reported in this paper are the mean of all three measures performed by each observer.

Statistical methods

Statistical analysis was completed using SPSS Version 25 (IBM Corp., Armonk, NY). Means, standard deviations, and 95% confidence intervals were calculated for continuous variables and frequencies were calculated for categorical variables. Intra-observer and inter-observer consistency were assessed using intraclass correlation (ICC) for continuous variables and Fleiss’ kappa for categorical variables. The optimal range of anterolateral fibula cortical contact length was determined by plotting contact length versus observer consistency of TFL distances.

## Results

Fifty-two ankles in 52 unique patients met the inclusion criteria. The measured anterolateral fibula cortical contact length ranged from a mean of 3.74 (95% CI, 3.24 to 4.24) mm to a mean of 11.45 (95% CI, 11.21 to 11.69) mm. The measured TFL distance ranged from a mean of -2.83 (95% CI, -3.65 to -2.00) mm to a mean of 5.03 (95% CI, 4.48 to 5.57) mm. For both anterolateral fibula cortical contact length (Table 1) and TFL distance measurements, intra-observer and inter-observer consistencies were excellent (minimum ICC, 0.87).

**Table 1:** Intraclass correlation (ICC) of linear distance measurements within ranges of linear distance

Group	Subjects	ICC
0 to 1.9	0	
2.0 to 3.9	1	
4 to 5.9	3	0.509
6 to 7.9	11	0.741
8 to 9.9	17	0.834
10 to 11.9	14	0.605
12 or more	0	

The mean TFL distance was less than 0 mm in 6 ankles (12%), between 0 and 1 mm in 19 ankles (37%), between 1 and 2 mm in 16 ankles (31%), and between 2 and 3 mm in eight ankles (15%). There was one ankle each (2%) between 3 and 4 mm, between 4 and 5 mm, and greater than 5 mm. For fibula morphology categorization, intra-observer consistency ranged from substantial to almost perfect (Fleiss’ Kappa range, 0.73 to 0.97) (Table 2). However, inter-observer consistency was only moderate (Fleiss’ Kappa, 0.55). The plot of mean anterolateral fibula cortical contact length versus ICC for TFL distance indicated an optimal range of 6 to 10 mm of fibula contact in order to maintain a minimum of excellent observer consistency (Table 3).

**Table 2:** Fibula morphology categorization reliability

Observer	Fleiss Kappa	95% CI	Interpretation of Agreement
1	0.97	0.79 to 1.00	Almost perfect
2	0.73	0.58 to 0.88	Substantial
3	1	0.60 to 1.00	Almost perfect
All	0.55	0.45 to 0.64	Moderate

**Table 3:** Interobserver intraclass correlation (ICC) of tibiofibular line (TFL) measurements by linear distance category

Group	Subjects	ICC All	Observer 1	Observer 2	Observer 3
0 to 1.9	0				
2.0 to 3.9	1				
4 to 5.9	3	0.382	0.812	0.779	0.881
6 to 7.9	11	0.803	0.979	0.797	0.974
8 to 9.9	17	0.942	0.981	0.947	0.961
10 to 11.9	14	0.655	0.948	0.615	0.75
12 or more	0				

## Discussion

Achieving proper anatomic syndesmosis reduction is important to decrease long-term morbidity after ankle fractures [8-10]. Despite this, ankle syndesmosis malreduction is alarmingly common and difficult to assess using traditional operative techniques [8,11,12]. Conventional radiography, the modality most commonly used to assess intraoperative syndesmosis reduction, is poorly suited to this task [3,13,14]. Multiple techniques for intraoperative syndesmosis evaluation have been proposed, but no gold standard has been identified [9,10,15-17]. 

The TFL technique was initially proposed by Gifford and Lutz [6]. Reb et al’s cadaveric model elaborated on this by demonstrating that the TFL could be translated into a visual intraoperative technique without the use of CT scans at all [7]. However, that study found relatively poor intra- and inter-observer reliability. The findings of this study refine and expand upon these works by showing that observer reliability varies by fibula morphology and that certain fibula anatomic variants had very high observer reliability. The clinical implication of this finding is that the TFL technique may be a reliable intraoperative tool in specific patients. This is encouraging because this technique introduces no cost, radiation, special instrumentation, or imaging. Overall, TFL measurements had excellent inter-observer and intra-observer reliability (minimum ICC, 0.87), and fibula morphology characterization showed at least substantial intra-observer reliability and moderate inter-observer reliability.

Among uninjured syndesmoses, a broader range of TFL values was observed than previously reported, including negative values, of which some were relatively large. Anterolateral fibula cortical contact length was more useful than fibula morphology in discerning which subjects were best suited for this technique. Excellent observer consistency occurred when 6 mm to 10 mm of fibula contact length was present. This linear length range of high consistency also represented 61% of the study population, suggesting the TFL technique may have high relevance in the majority of patients. Surgeons considering the technique should be cognizant of the fibula cortical contact length, which can either be measured intraoperatively or on a preoperative CT scan if one is obtained.

While morphology characterization had substantial to almost perfect intra-observer reliability, inter-observer reliability was moderate. However, anterolateral fibula cortical contact length was most relevant in regard to TFL measurement reliability correlation. This indicates the consistency of linear cortical contact length was more important than the consistency of morphology categorization for the purpose of this technique. As a result, reliable linear contact length measurement may be important during the operative approach; proper identification of a linear fibula in the ideal 6-10 mm range may prompt posterolaterally positioned plate and screws so as to allow intraoperative utilization of the TFL technique at the open anterolateral fibula surface.

The negative values raise questions regarding TFL validity across a broad spectrum of anatomical variation and injury modalities. While these values could be a result of observer error or limitations of the measurement software itself, they more likely represent a somewhat common normal anatomic variant in certain subjects, suggesting further challenges related to morphology beyond reliability. Concave, convex, or internally rotated linear fibulas yielding negative measurements on CT would be physically limited in an intraoperative context due to the ruler being unable to pass into the bone to record negative measurements. As a result, further characterization of the context and prevalence of conditions producing a negative TFL is warranted to fully assess TFL viability in this minority.

While we believe intraoperative utilization of the TFL can be a valuable adjunct to visually assess syndesmotic reduction, surgeons should be cognizant to not solely rely on this technique. Certain combinations of fibula orientation may lead to a normal-appearing TFL in the presence of syndesmotic malreduction. For example, fibular external rotation is expected to increase TFL magnitude while posterior fibula displacement is expected to decrease TFL magnitude. A combination of the two could effectively cancel each other out, producing a TFL indicating a reduced state despite the actual presence of malreduction. This may account for Marmor et al’s findings of unreliable fluoroscopic reduction assessment in the context of external fibular rotation [18]. TFL reliability may need to be assessed in the context of these various orientations, with priority placed on the most common orientations of injury found in the patient population.

Linear fibula cortical length was shown to play a critical role in determining the ideal context for the employment of the TFL technique in syndesmosis reduction. Overall, the TFL technique may be best suited for assessing syndesmosis reduction among patients with between 6 and 10 mm of straight anterolateral fibula cortex upon which to base the TFL. These results support the validity of the TFL technique and further characterize its role in the clinical setting. Given the value of linear cortical length, future studies could consider other heights above the tibial plafond besides 10 mm, as alternatives may provide longer axial fibular lengths more often, thus increasing the percentage of patients amenable to this technique.

This study has several limitations. While our sample size of 52 was adequately powered for statistical analysis, a larger cohort may improve external validity and generalizability. Each of our three observers assessed each patient multiple times separated by large periods of time (weeks to months) to decrease the bias of short-term recall, but it is still possible that certain patients or measurements were remembered between sessions. The three observers have different levels of medical training and experience with CT scans, which may have impacted measurement accuracy. As previously discussed, further research is needed to assess the intraoperative utility of the TFL in patients with negative TFL measurements.

## Conclusions

Intraoperative use of the TFL can be a valuable adjunct, allowing surgeons to visually assess syndesmotic reduction without introducing additional cost, radiation, imaging, or instrumentation. This technique appears best suited to patients with between 6 mm and 10 mm of straight anterolateral fibula cortex upon which to base the TFL, which the majority of evaluated patients had. Measurement of the TFL and anterolateral fibula cortical contact length have high inter-observer and intra-observer reliability. Characterization of fibula morphology has high intra-observer reliability and moderate inter-observer reliability.

## References

[1] Jones MH, Amendola A (2007). Syndesmosis sprains of the ankle. A systematic review. Clin Orthop Relat Res.

[2] Mak MF, Stern R, Assal M (2018). Repair of syndesmosis injury in ankle fractures: current state of the art. EFORT Open Rev.

[3] Krähenbühl N, Weinberg MW, Davidson NP, Mills MK, Hintermann B, Saltzman CL, Barg A (2018). Imaging in syndesmotic injury: a systematic literature review. Skeletal Radiol.

[4] Thordarson DB, Motamed S, Hedman T, Ebramzadeh E, Bakshian S (2019). The effect of fibular malreduction on contact pressures in an ankle fracture malunion model. J Bone Joint Surg Am.

[5] Williams GN, Jones MH, Amendola A (2007). Syndesmotic ankle sprains in athletes. Am J Sports Med.

[6] Gifford PB, Lutz M (2014). The tibiofibular line: an anatomical feature to diagnose syndesmosis malposition. Foot Ankle Int.

[7] Reb CW, Hyer CF, Collins CL, Fidler CM, Watson BC, Berlet GC (2016). Clinical adaptation of the “tibiofibular line” for intraoperative evaluation of open syndesmosis reduction accuracy: a cadaveric study. Foot Ankle Int.

[8] Sagi HC, Shah AR, Sanders RW (2012). The functional consequence of syndesmotic joint malreduction at a minimum 2-year follow-up. J Orthop Trauma.

[9] Schreiber JJ, McLawhorn AS, Dy CJ, Goldwyn EM (2013). Intraoperative contralateral view for assessing accurate syndesmosis reduction. Orthopedics.

[10] Summers HD, Sinclair MK, Stover MD (2013). A reliable method for intraoperative evaluation of syndesmotic reduction. J Orthop Trauma.

[11] Gardner MJ, Demetrakopoulos D, Briggs SM, Helfet DL, Lorich DG (2006). Malreduction of the tibiofibular syndesmosis in ankle fractures. Foot Ankle Int.

[12] Gonzalez T, Egan J, Ghorbanhoseini M (2017). Overtightening of the syndesmosis revisited and the effect of syndesmotic malreduction on ankle dorsiflexion. Injury.

[13] van den Heuvel SB, Dingemans SA, Gardenbroek TJ, Schepers T (2019). Assessing quality of syndesmotic reduction in surgically treated acute syndesmotic injuries: a systematic review. J Foot Ankle Surg.

[14] Nielson JH, Gardner MJ, Peterson MG, Sallis JG, Potter HG, Helfet DL, Lorich DG (2005). Radiographic measurements do not predict syndesmotic injury in ankle fractures: an MRI study. Clin Orthop Relat Res.

[15] Dubois-Ferrière V, Gamulin A, Chowdhary A, Fasel J, Stern R, Assal M (2016). Syndesmosis reduction by computer-assisted orthopaedic surgery with navigation: feasibility and accuracy in a cadaveric study. Injury.

[16] Lee JY, Lim JH, Jung GH (2018). Radiological indicator of reduction adequacy during ankle syndesmosis surgery: a computational cadaveric study. Injury.

[17] Ruan Z, Luo C, Shi Z, Zhang B, Zeng B, Zhang C (2011). Intraoperative reduction of distal tibiofibular joint aided by three-dimensional fluoroscopy. Technol Health Care.

[18] Marmor M, Kandemir U, Matityahu A, Jergesen H, McClellan T, Morshed S (2013). A method for detection of lateral malleolar malrotation using conventional fluoroscopy. J Orthop Trauma.

